# Screening Gene Mutations in Chinese Patients With Benign Essential Blepharospasm

**DOI:** 10.3389/fneur.2019.01387

**Published:** 2020-01-23

**Authors:** Hongjuan Dong, Ying Luo, Shanghua Fan, Bo Yin, Chao Weng, Bin Peng

**Affiliations:** Department of Neurology, Renmin Hospital of Wuhan University, Wuhan, China

**Keywords:** benign essential blepharospasm, Chinese, gene mutation, SYNE1, CIZ1

## Abstract

**Objective:** This study aimed to screen gene mutations in Chinese patients with benign essential blepharospasm (BEB) to understand its etiology.

**Methods:** Twenty BEB patients diagnosed by clinical manifestations between April 2015 and October 2015 were enrolled. All the cases were investigated by questionnaires about general conditions, social behavioral factors, environmental factors, psychological factors, genetic factors, and previous diseases. In each patient, a total of 151 genes related to movement disorders were analyzed by second-generation sequencing.

**Results:** Two patients had a family history of BEB, and they had SYNE1 and Cdkn1A-interacting zinc finger protein 1 (CIZ1) mutation, respectively. We found the SYNE1 mutation in seven patients, the CIZ1 mutation in two patients, the CACNA1A mutation in two patients, the LRRK2 mutation in two patients, and the FUS mutation in two patients. The C10orf2, TPP1, SLC1A3, PNKD, EIF4G1, SETX, PRRT2, SPTBN2, and TTBK2 mutations were found in only one patient, respectively, while not any mutation in the 151 genes were found in two patients. Some patients had mutations in two genes.

**Conclusion:** Genetic factors, especially SYNE1 and CIZ1 mutations, contribute to the etiology of BEB.

## Introduction

Blepharospasm is an adult-onset focal dystonia characterized by involuntary contraction of the orbicularis oculi muscles. Benign essential blepharospasm (BEB) is the most common form of blepharospasm, and its etiology remains elusive (1). Studies in various populations suggest that BEB has a prevalence of 12–133 cases per million (2). Most cases of BEB are considered sporadic; about 20–30% of cases have a family history. Epidemiological survey showed that BEB was more common in women than in men with a 2.3:1 ratio (3).

The average age of onset in blepharospasm patients is in the 50s, and the onset for the female is 4.7 years late compared with the male (4). Initially, the spasms are mild and infrequent, but symptoms typically progress and are highly disruptive to the patient's visual activities and quality of life (4). While some BEB patients have family history, the disease gene has not been determined. In familial BEB, DYT1, and other dystonia-related genes were detected, but no definitive pathogenic genes have been identified (5, 6). Therefore, in this study we aimed to screen gene mutations in Chinese BEB patients to understand its genetic etiology. We performed genetic testing in 20 BEB patients on 151 genes related to movement disorders, including 27 genes related to dystonia, 4 genes related to episodic ataxia (EA), 2 genes related to paroxysmal kinesigenic dyskinesia (PKD), 1 gene related to paroxysmal non-kinesigenic dyskinesia (PNKD), 3 genes related to essential tremor, 78 genes related to Parkinson's disease, and PDK3, AFG3L2, and ANO10 (7, 8). We also analyzed the relationship between clinical manifestations and gene mutations in the 20 patients.

## Methods

### Patients

This study was approved by the Medical Ethics Committee of Renmin Hospital of Wuhan University. Written informed consent was obtained from each patient. Twenty patients with BEB, who were referred to the Movement Disorders Clinic in the Department of Neurology at our hospital between April 2015 and October 2015, were evaluated through clinical examination and questionnaire. Each patient had neurological examination by a movement disorder specialist and completed a questionnaire. All patients had a full neurological examination to rule out other underlying neurological conditions.

The questionnaire was divided into four parts: general characteristics (onset time, age of onset, initial site, sensory tricks, first visit time, the time of confirmed diagnosis, blood pressure), past and family history (hypertension, diabetes, heart disease, hyperthyroidism, measles, cerebrovascular disease, brain trauma, encephalitis, ocular diseases), medicine history, and dangerous factors (pesticides, herbicides, preservatives, living environment, smoking and drinking, drug abuse).

### Sequencing

Genomic DNA was extracted from the blood of each patient using Flexi DNA Kit (QIAGEN, Germany), which was sent to Kangso Medical Inspection (Beijing, China) for next-generation sequencing for genes listed in Table 1.

**Table 1:** The genes analyzed in this study.

**Disease**	**Number of genes**	**Genes**
Dystonia	27	ACTB, TAF1, PANK2, SLC6A3, ANO3, FA2H, PARK2, SPR, ARX, FOXG1, PLA2G6, TH, ATP1A3, GCH1, PRKRA, THAP1, BCAP31, GNAL, SERAC1, TOR1A, CIZ1, MECP2, SGCE, TUBB4A, DRD2, OPA3, SLC30A10
EA	4	CACNA1A, KCNA1, SLC1A3, DARS2
PKD	2	PRRT2, SLC2A1
PNKD	1	PNKD
ET	3	FUS, MAPT, LINGO1
Parkinson's disease	78	ANG, DNAJC6, HTRA2, PRKRA, APOE, DRD1, IREB2, PSEN1, APP, DRD2, LAMP2, PSEN2, ATG7, DRD3, LRRK2, RAB7L1, ATP13A2, DRD4, LTF, SLC30A10, ATP1A3, EIF4G1, MAOA, SLC6A3, ATP6AP2, FBXO7, MAPT, SLC6A4, BDNF, FMR1, MTHFR, SMPD1, BST1, FOXG1, MTR, SNCA, CBS, FTH1, MTRR, SPG11, CCK, GAK, NEFM, SV2C, CCKAR, GBA, NR4A2, SYNE1, CNR1, GBAP1, PARK2, TEF, COMT, GDNF, PARK7, TFRC, CP, GIGYF2, PARL, TH, CRY1, GRIN2A, PIK3CD, TRPM2, CYP27A1, GRN, PINK1, UCHL1, DCTN1, HFE, PLA2G6, VPS35, DGKQ, HOMER1, POLG, DNAJC13, HTR2A, PRKAG2
ADCK3, AFG3L2, ANO10, and so on	36	ADCK3, AFG3L2, ANO10, ATCAY, ATP2B3, ATP8A2, C10orf2, CA8, STUB1, DNMT1, EEF2, FGF14, GBA2, GJB1, GRID2, GRM1, ITPR1, KCNA1, KCNC3, KCND3, NUBPL, PDYN, PEX2, PLEKHG4, PRKCG, SACS, SETX, SPTBN2, SRD5A3, SYT14, TDP1, TGM6, TPP1, TTBK2, VLDLR, ZNF592

## Results

BEB was more common in women than in men. This study included 17 women and 3 men. The mean age was 55.25 years (range 31–72), and the mean duration of illness was 6.15 years (range 1–24). The early symptoms of the 20 patients were different. Nine patients had difficulty of opening the eyes, seven patients showed increased blinking, two patients presented ocular muscle weakness, and two patients showed eyelid twitching.

A total of 15 patients chose to first consult an ophthalmologist. Only five patients were correctly diagnosed at the first counseling. From the first visit to the diagnosis, most patients had visited three hospitals. The mean time for intervals between initial symptoms to definitive diagnosis was 4.11 years; one patient had symptoms for 15 years before a formal diagnosis was given.

One patient had carbon monoxide poisoning, three patients had a history of hypertension, and one patient had hypertension, diabetes, cerebrovascular disease, and ovariectomy. Two patients were smokers. Three patients had a history of drinking. Five patients had a history of exposure to pesticides/herbicides. Four patients were tea drinkers. Four patients had a history of exposure to cement. One patient reported taking estrogen drugs.

For treatment, all patients received botulinum toxin (BoNT). In most patients, the symptoms improved with BoNT but did not completely resolve. Seven patients received myectomy procedure. In addition, other treatments such as acupuncture were used but had no obvious benefits.

**Figure 1:**
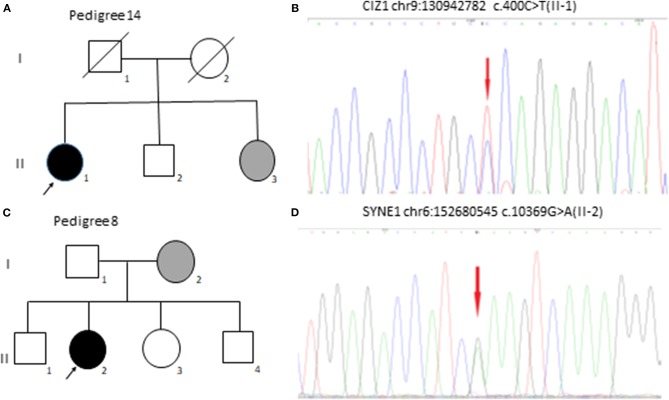
CIZ1 and SYNE1 variants in pedigree 14 and pedigree 8 with BEB. **(A)** Family 14 with BEB. One affected family member's (II-1) sister (II-3) was diagnosed with BEB but did not do genetic testing. **(B)** Electropherograms of II-1 was heterozygous for CIZ1 (chr9: 130942782 c.400C>T). **(C)** Family 8 with BEB. One affected family member's (II-2) mother (I-2) was diagnosed with BEB but did not do genetic testing. **(D)** Electropherograms of II-2 was heterozygous for SYNE1 (chr6: 152680545 c.10369G>A).

Two patients reported a family history of BEB. For the 151 genes listed in Table 1, we detected SYNE1 gene mutation in seven cases, Cdkn1A-interacting zinc finger protein 1 (CIZ1) gene mutation in two cases, CACNA1A gene mutation in two cases, LRRK2 gene mutation in two cases, FUS gene mutation in two cases, and C10orf2, TPP1, SLC1A3, PNKD, EIF4G1, SETX, PRRT2, SPTBN2, and TTBK2 gene mutation each in one case. Two patients did not have any mutations in the 151 genes. The clinical data and mutation genes of 20 patients with BEB are presented in Table 2. Representative CIZ1 and SYNE1 variants in pedigrees and mutation analysis are shown in Figure 1.

**Table 2:** Clinical data and mutation genes of 20 patients with BEB.

**Patient number**	**Gender**	**Age (years)**	**Family history**	**Duration (years)**	**Mutation genes**	**Heterozygous variant**	
1	F	55	N	6	PARK2 CACNA1A	c.652T>A c.62C>T	p.Ser218Thr p.Ala21Val
2	F	44	N	3	SETX PRRT2	c.389-9delT c.236>T	- p.Ser79Leu
3	F	63	N	1	SPTBN2 FUS	c.5939C>A c.1164G>A	p.Ala1980Glu p.Met388Ile
4	F	52	N	8	SYNE1 FUS	c.20677C>A c.776A>G	p.Gln6893Lys p.Asn259Ser
5	F	65	N	8	SYNE1 EIF4G1	c.5438A>G c.1816C>T	p.His1813Arg p.Pro606Ser
6	F	52	N	10	SYNE1 CACNA1A	c.11968C>G c.6476G>A	p.Pro3990Ala p.Arg2159His
7	F	46	N	4	SYNE1 CIZ1	c.17438C>G c.2380C>T	p.Pro5813Arg p.Arg794Cys
8	F	47	Y	4	SYNE1	c.10369G>A	p.Glu3457Lys
9	M	31	N	1	SYNE1	c.23999G>A	p.Arg8000His
10	M	52	N	4	SYNE1	c.13072G>A	p.Asp4358Asn
11	F	62	N	6	LRRK2	c.6448G>A	p.Val2150Ile
12	F	63	N	2	LRRK2	c.5078G>A	p.Arg1693Gln
13	M	57	N	2	C10orf2	c.1495G>T	p.Asp499Tyr
14	F	70	Y	10	CIZ1	c.400C>T	p.Pro134Ser
15	F	52	N	1	PNKD	c.301C>T	p.Arg101Trp
16	F	72	N	24	SLC1A3	c.985G>A	p.Ala329Thr
17	F	52	N	5	TPP1	c.1681C>T	p.Leu561Phe
18	F	52	N	12	TTBK2	c.3290T>C	p.Val1097Ala
19	F	59	N	6	-		
20	F	59	N	6	-		

## Discussion

The etiology of BEB is still unclear. It was speculated that the pathogenesis of BEB may be related to basal ganglia damage and the disorders of neurotransmitters such as dopamine (9–11). The abnormality of dopaminergic pathways may be implicated in the pathogenesis of dystonia (11). In particular, BEB patients had reduced number of nerves in the sub-basal plexus, which suggested that the impairment in corticosensory processing due to the defect of the sensorimotor gating mechanism could cause the loss of inhibition of blink reflex (12).

Genetic factors play an important role in neurological diseases (13). There is evidence for an autosomal-dominant gene with reduced penetrance contributing to BEB (14). Therefore, in this study we examined 151 genes in all BEB patients and found SYNE1 gene mutation in seven cases, CIZ1 gene mutation in two cases, CACNA1A gene mutation in two cases, LRRK2 gene mutation in two cases, FUS gene mutation in two cases, and C10orf2, TPP1, SLC1A3, PNKD, EIF4G1, SETX, PRRT2, SPTBN2, and TTBK2 gene mutation in each case. Interestingly, a recent study showed that deleterious variants in CACNA1A, REEP4, TOR2A, ATP2A3, HS1BP3, GNA14, and DNAH17 were involved in blepharospasm, and none of these variants except for CACNA1A has been reported to be associated with blepharospasm (15). However, a follow-up study reported that all variants detected in GNAL, CIZ1, and TOR2A seemed to be benign in 132 blepharospasm patients (16). Therefore, we should investigate the status of these variants in our patients in further studies.

SYNE1 gene encodes nesprin-1 protein and genome-wide association studies have revealed the strong association of SYNE1 gene with bipolar disorder and major depression (17). In particular, a recent study showed that genetic variation in the CPG2 region of SYNE1 could be linked to glutamatergic synapse dysfunction and the susceptibility to bipolar disorder (18). While BEB is commonly associated with emotional disorders, to our knowledge, no direct association between SYNE1 and BEB has been reported in the literatures. In this study, we found the highest number of patients that had gene mutations in SYNE1 gene, suggesting that SYNE1 may play a role in the pathogenesis of BEB. CIZ1 was first identified as a protein that interacted with CDK2 inhibitor p21. CIZ1 gene localizes on 9q34, and the encoded protein CIZ1 is comprised of 842 amino acid residues and contains two glutamine-rich domains, three C2H2-type zinc finger motifs, an acidic domain, and an MH3 domain. CIZ1 is crucial for the initiation of DNA replication and genomic DNA integrity (19). In this study, two patients had CIZ1 gene mutations, consistent with previous studies confirming that CIZ1 mutations were related to the pathogenesis of dystonia (20, 21), but not in agreement with a recent study showing no CIZ1 mutations in blepharospasm patients (16).

Interestingly, in this study only two patients reported a family history of BEB, and SYNE1 and CIZ1 gene mutations were detected in each of these two patients, respectively. These results strongly suggest that SYNE1 and CIZ1 gene mutations are the main genetic factors that contribute to the pathogenesis of BEB. However, this study has several limitations. Firstly, the total number of patients is small. Secondly, several potential new genes associated with BEB have been reported recently, and they were not included in the genes that we screened in our study. Further studies are needed to understand the pathogenic mechanism of BEB in Chinese patients.

## Data Availability Statement

All datasets generated for this study are included in the article/supplementary material.

## Ethics Statement

This study was approved by Medical Ethics Committee of Renmin Hospital of Wuhan University. Written informed consent was obtained from each patient. The patients/participants provided their written informed consent to participate in this study.

## Author Contributions

BP contributed to the design of the study. HD and YL contributed to manuscript drafting. SF and BY in collected and analyzed the samples. CW helped to analysis the data and draw the figure and revised the paper. All authors finally approved the version to be published and agree to be accountable for all aspects of the work.

### Conflict of Interest

The authors declare that the research was conducted in the absence of any commercial or financial relationships that could be construed as a potential conflict of interest.
